# Factors during training which predict future use of minimally invasive thoracic surgery

**DOI:** 10.1016/j.amsu.2018.09.039

**Published:** 2018-10-01

**Authors:** Paul E. Rothenberg, Byron D. Hughes, Farshad Amirkhosravi, Bless P. Onaiwu, Ikenna C. Okereke

**Affiliations:** a School of Medicine, University of Texas Medical Branch, Galveston, TX, USA; b Department of Surgery, University of Texas Medical Branch, Galveston, TX, USA; c Division of Cardiothoracic Surgery, University of Texas Medical Branch, Galveston, TX, USA

**Keywords:** Esophagectomy, Lobectomy, Minimally invasive surgery thoracic surgery, Video-assisted thoracoscopic surgery (VATS)

## Abstract

**Background:**

While minimally invasive thoracic surgery (MIS) has increased nationwide over the years, most patients undergoing lung and esophageal resections still undergo an open approach. We performed a national survey to analyze factors associated with a propensity to perform MIS after completing a cardiothoracic training program.

**Materials and methods:**

Cardiothoracic surgery trainees in 2 or 3-year programs from 2010 to 2016 were sent an online survey regarding the numbers and types of cases performed during training and current practice patterns as attending surgeons. Comfort level with MIS was also assessed. Responses were recorded and analyzed using SPSS.

**Results:**

One hundred thirty-six trainees responded, with a mean of 121 lobectomies (30-250) and 40 esophagectomies (8-110) performed during training. Mean minimally invasive lobectomy and esophagectomy rates during training were 53% and 30% respectively. A greater ratio of MIS procedures performed during training correlated with a higher rate performed as an attending (lobectomies, p = 0.04; esophagectomies, p = 0.01) and a greater comfort level with performing these procedures (lobectomies, p = 0.01 and esophagectomies, p < 0.01).

**Conclusions:**

Based on these results, performing a greater ratio of minimally invasive lobectomies and esophagectomies during fellowship training increases the likelihood of performing them as an attending.

## Highlights

• Trainees who perform more minimally invasive procedures as trainees are more likely to do so as attendings.
• Trainees who perform many open lobectomies do not necessarily convert to minimally invasive approaches as attendings.
• Trainees who perform many esophagectomies are less likely to convert as attendings.

## 1 Introduction

There has been a lag in adoption and full implementation of minimally invasive surgery in cardiothoracic surgery. While minimally invasive thoracic surgery has been associated with faster recovery, lower morbidity and mortality, decreased length of stay (LOS), and costs, the national utilization rate of minimally invasive approaches in thoracic surgery has remained at approximately 40% [1].

One reason that the implementation of minimally invasive techniques in thoracic surgery has been slow is the lack of confidence in these approaches by residents and fellows at the end of their training. A previous study reported the confidence level of recent thoracic surgery graduates to be 56% and 46% for performing minimally invasive pulmonary and esophageal operations respectively [2]. Understanding the causes of low confidence levels for some recent graduates would help to identify possible training insufficiencies and allow programs to adapt as needed. The goal of this study was to determine the factors in cardiothoracic education which were associated with increased use of minimally invasive approaches after training.

## 2 Materials and Methods

### 2.1 Subjects

All standard 2 or 3-year cardiothoracic surgery training programs in the United States were included. After obtaining institutional review board approval, contact information was obtained for every trainee (n = 608) who had finished from 2010 to 2016. Contact information was obtained from the Thoracic Surgery Directors Association (TSDA) and the Cardiothoracic Surgery Network (CTSNet). Participation was voluntary.

### 2.2 Survey

**Fig. 1:**
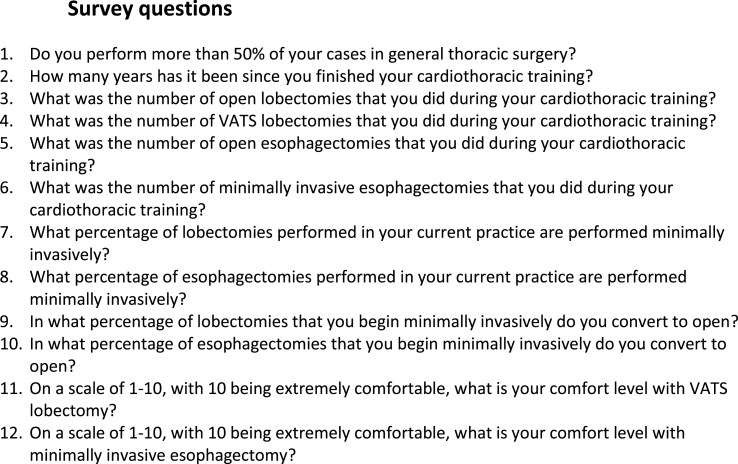
Survey questions.

A 12-question survey was constructed by the authors (Fig. 1). The survey asked trainees to recall the number of lobectomies and esophagectomies performed, and the percentage of each which were performed minimally invasively. The survey also asked trainees to provide their current practice patterns as attendings and their comfort level with minimally invasive lobectomy and minimally invasive esophagectomy (MIE). The comfort level scale ranged from 1 (very uncomfortable) to 10 (completely comfortable).

To create the survey, each of the cardiothoracic surgeons at our institution was queried about factors which they felt contributed to a propensity to perform minimally invasive surgery as an attending. Multiple general surgeons at our institution were also queried, to decrease any potential bias of cardiothoracic surgery alone and because our general surgery team has a high overall rate of usage of minimally invasive techniques. The overall aggregate of responses were used to create the topics for the 12 questions.

To design each question, previous literature on survey design in education research was used to craft each question [3]. Specifically, the manner used to create the questions was based on a previously validated process for developing questionnaires in medical education research [4].

### 2.3 Survey administration

A survey was emailed to each trainee. Each email outlined the project, details of anonymity and the opportunity to be awarded. An incentive $50 online cash card was given randomly to 10 respondents.

### 2.4 Statistical analysis

Survey information was analyzed using SPSS (Version 24). One-way analysis of variance and independent samples t-testing were used to determine statistically significant associations of different variables. To analyze the association of number/percentage of surgeries versus the percentage of minimally invasive usage as attending surgeons, the number of lobectomies/esophagectomies was used as a continuous value without creating a cutoff value.

## 3 Results

There was a response rate of 22.4% (136/608). Graduates had performed an average of 121 lobectomies and 40 esophagectomies during their training (Table 1). Fifty-two percent of lobectomies and 30% of esophagectomies during training were performed minimally invasively.

**Table 1:** Survey responses of recent cardiothoracic surgery graduates (2010-2016).

	Mean
**Lobectomy**	
Open lobectomies performed during training	58 (4–250)
Minimally invasive lobectomies performed during training	63 (0–250)
Conversion rate of minimally invasive lobectomies as attending	12% (5–100)
Mean comfort level with minimally invasive lobectomy as attending	7.4 (1–10)

**Esophagectomy**	
Open esophagectomies performed during training	28 (1–150)
MIEs performed during training	12 (0–110)
Conversion rate of MIEs as attending	16% (5–100)
Mean comfort level with MIEs as attending	5.1 (1–10)

As attendings, the overall group currently performed 68% of their lobectomies minimally invasively. The mean comfort level was 7.4. Their mean conversion rate was 12%. Trainees were more likely to perform lobectomies minimally invasively if they had performed a higher number of minimally invasive lobectomies (p < 0.01) and a higher ratio of minimally invasive lobectomies versus all lobectomies (p < 0.01) during training (Table 2). Furthermore, the comfort level of minimally invasive lobectomy as an attending was associated with a higher number of minimally invasive lobectomies (p < 0.01) and a higher ratio of minimally invasive lobectomies versus all lobectomies (p < 0.01) during training. The likelihood of performing minimally invasive lobectomies as an attending was not associated with the number of years since the end of training (p = 0.71) or the total number of lobectomies completed as a trainee (p = 0.07). Further, the VATS mean conversion rate was 12% and had no association between the number of years since the end of fellowship (p = 0.49).

**Table 2:** Association of factors during training with lobectomy practice pattern as attending. p-values are shown.

	Years post-training	Number of minimally invasive lobectomies as trainee	Number of total lobectomies as fellow	Percentage of minimally invasive lobectomies as trainee
Percentage of lobectomies performed minimally invasively as an attending	0.71	<**0.01**	0.06	<**0.01**
Minimally invasive lobectomy comfort level	0.86	<**0.01**	0.13	<**0.01**

The overall group currently performed 40% of their esophagectomies minimally invasively. The mean comfort level was 5.1. Their mean conversion rate was 16%. Both the likelihood of performing MIE and the comfort level with MIE as attendings were associated with a higher number of total esophagectomies in training (p < 0.01), a higher number of MIEs in training (p < 0.01) and a higher ratio of MIEs versus all esophagectomies in training (p < 0.01) (Table 3). The mean conversion rate for MIE was 16%. The number of years since the completion of fellowship and the conversion rate was not statistically significant (p = 0.95).

**Table 3:** Association of factors during training with esophagectomy practice pattern as attending. p-values are shown.

	Years post-training	Number of MIEs as trainee	Number of total esophagectomies as trainee	Percentage of MIEs as trainee
Percentage of esophagectomies performed minimally invasively as an attending	0.69	<**0.01**	<**0.01**	<**0.01**
MIE comfort level	0.31	<**0.01**	<**0.01**	<**0.01**

## 4 Discussion

The prevalence of minimally invasive surgery has increased across most surgical disciplines over time [[2], [3], [4], [5]]. The benefits of minimally invasive approaches in thoracic surgery have been documented in the literature, and include a decrease in LOS, pain levels and perioperative pulmonary complications [[6], [7], [8]]. As such, there would be a benefit to public health with an increased frequency of minimally invasive thoracic procedures [9]. Minimally invasive resections have been adopted much more quickly in other fields, such as prostatectomy or cholecystectomy, than in thoracic surgery. The risk of pulmonary artery injury and the complexity of minimally invasive esophageal resection have likely been the major reasons for the slow adoption rate in thoracic surgery. It is encouraging, though, that recent graduates were performing close to 70% of their pulmonary resections using a minimally invasive approach. This rate is much higher than the overall national rate of 40%.

Our study showed that graduates who performed more minimally invasive procedures during their training were more likely to adopt similar behavior as attendings. Though this association may seem intuitive, it was interesting that performing a large number of open pulmonary resections as a trainee was not associated with increased ease in transitioning to a minimally invasive approach as an attending. It has previously been assumed that it is important for the trainee to “learn the anatomy” of the pulmonary hilum, after which adopting minimally invasive approaches to pulmonary resection would become easier. Our study suggests, however, that trainees who are predominantly exposed to open techniques to pulmonary resection may be less willing or able to use minimally invasive techniques as an attending. It is likely that this phenomenon occurs because trainees are more able to modify their practice pattern before fixed “habits” develop by the end of their training. As such, we would favor increased exposure of trainees to minimally invasive pulmonary resections during their training.

Our study revealed that the adoption of MIE as an attending was associated with the overall number of esophagectomies, open and minimally invasive, performed as a trainee. We feel that this trend was seen because esophagectomy is much less likely to have a catastrophic intraoperative complication, such as bleeding, than lobectomy. As such, trainees with significant esophageal experience may be more comfortable with the procedure and less hesitant to attempt a minimally invasive approach as an attending.

As with other minimally invasive techniques, there is a learning curve that must be achieved before obtaining proficiency. Our study results indicate that the curricula of thoracic surgery fellowship programs which incorporate the use of minimally invasive surgery technique appear to be more important when compared to the years since the completion of fellowship for adoption of these methods. The learning curve for minimally invasive lobectomy has been reported to be 50 cases [6,10]. MIE generally has more steps involved and is a longer operation than a lobectomy, but has been increasingly used as an alternative to an open approach [9,11,12]. One study reported that 14% of surveyed surgeons preferred MIE as a main method of esophagectomy [13]. It appears that prevalence of MIE is increasing with time, as the recent graduates in our study were performing 40% of their esophagectomies using a minimally invasive approach. In comparison to lobectomies, the learning curve to reduce technical complications is steeper. According to one study, approximately 119 cases are needed to reduce a surgeon's anastomotic leak rate [[14], [15], [16], [17]].

There were some limitations of this study. Firstly, our response rate was only 22.4%. For future implementation of similar studies, partnering with national organizations such as the American Association for Thoracic Surgery (AATS) to distribute at the national meeting may augment the response rate. This would allow access to a larger population which would likely increase our sample size of recent graduates who completed the survey. Secondly, there could have been recall bias from the respondents who have been out of training for a longer period of time. However, email surveys are an optimal method to obtain data and preserve anonymity among respondents [18]. Lastly, our survey did not include factors which may have a role in our outcomes such as additional training (i.e. minimally invasive fellowship), 2-year versus 3-year fellowship programs [19], and current institutional facilities as an attending. The authors believe gathering this specific information for each respondent could have made it difficult to ensure anonymity of the surgeon agreeing to complete the surgery. Despite these potential limitations, our study provides new information on the impact of cardiothoracic surgery training on future MIS adoption in thoracic surgery.

## 5 Conclusions

Increased exposure to minimally invasive techniques during cardiothoracic training will better prepare trainees to overcome the learning curves of both procedures, and they will be more likely to incorporate minimally invasive surgery into their practices as attendings. Performing a majority of procedures while in training using an open approach may decrease the probability of adopting a minimally invasive approach as an attending, especially for lobectomy.

## Author disclosures

None.

## Funding

Dr. Hughes is supported by a grant from the National Institute of Diabetes and Digestive and Kidney Diseases of the National Institutes of Health (T32DK007639). The content is solely the responsibility of the authors and does not necessarily represent the official views of the National Institutes of Health.

## Provenance and peer review

Not commissioned, externally peer reviewed.

## Ethical approval

Institutional Review Board approval was given for this study.

The reference number is 17–0062.

## Sources of funding

The funding was departmental.

## Author contribution

Farshad Amirkhosravi contributed to the writing and development of the manuscript. Bless Onaiwu contributed to the development of the manuscript. Byron Hughes was responsible for the organization, writing and development of this manuscript. Paul Rothenberg gathered and organized the list of graduates. Paul Rothenberg and Dr. Okereke developed the email survey. Dr. Okereke was responsible for the overall supervision of the study.

## Conflicts of interest

There are no conflicts of interest for any of the authors.

## Research registration number

The research has been registered with the Chinese Clinical Trial Registry. The review number is 17–0062.

## Guarantor

Ikenna Okereke, MD.

Associate Professor of Surgery.

Chief, Thoracic Surgery.

University of Texas Medical Branch.

301 University Blvd.

Galveston, TX 77555.

(409) 772-0534

Fax (409) 772-5611

ikokerek@utmb.edu.

## Consent

N/A.

## References

[1] Shigemura N., Akashi A., Funaki S. (2006). Long-term outcomes after a variety of video-assisted thoracoscopic lobectomy approaches for clinical stage IA lung cancer: a multi-institutional study. J. Thorac. Cardiovasc. Surg..

[2] Chu D., Vaporciyan A.A., Iannettoni M.D. (2016). Are there gaps in current thoracic surgery residency training programs?. Ann. Thorac. Surg..

[3] Amore D., Curcio C. (2017). Steps in the development of a VATS lobectomy program. J. Vis. Surg..

[4] Cao C., Petersen R.H., Yan T.D. (2014). Learning curve for video-assisted thoracoscopic lobectomy. J. Thorac. Cardiovasc. Surg..

[5] Yan T.D., Cao C., D'Amico T.A. (2014). Video-assisted thoracoscopic surgery lobectomy at 20 years: a consensus statement. Eur. J. Cardio. Thorac. Surg..

[6] McKenna R.J. (2008). Complications and learning curves for video-assisted thoracic surgery lobectomy. Thorac. Surg. Clin..

[7] Bedetti B., Bertolaccini L., Solli P., Scarci M. (2017). Learning curve and established phase for uniportal VATS lobectomies: the Papworth experience. J. Thorac. Dis..

[8] Sandri A., Filosso P.L., Lausi P.O., Ruffini E., Oliaro A. (2016). VATS lobectomy program: the trainee perspective. J. Thorac. Dis..

[9] Cheng X., Onaitis M.W., D'Amico T.A., Chen H. (2018). Minimally invasive thoracic surgery 3.0: lessons learned from the history of lung cancer surgery. Ann. Surg..

[10] Gonfiotti A., Bongiolatti S., Borgianni S. (2016). Development of a video-assisted thoracoscopic lobectomy program in a single institution: results before and after completion of the learning curve. J. Cardiothorac. Surg..

[11] Ruurda J.P., van der Sluis P.C., van der Horst S., van Hilllegersberg R. (2015). Robot-assisted minimally invasive esophagectomy for esophageal cancer: a systematic review. J. Surg. Oncol..

[12] Sudarshan M., Ferri L. (2012). A critical review of minimally invasive esophagectomy. Surg. Laparosc. Endosc. Percutaneous Tech..

[13] Boone J., Livestro D.P., Elias S.G., Borel Rinkes I.H., van Hillegersberg R. (2009). International survey on esophageal cancer: part I surgical techniques. Dis. Esophagus.

[14] Tapias L.F., Morse C.R. (2014). Minimally invasive Ivor Lewis esophagectomy: description of a learning curve. J. Am. Coll. Surg..

[15] van Workum F., Stenstra M., Berkelmans G.H.K. (2017). Learning curve and associated morbidity of minimally invasive esophagectomy: a retrospective multicenter study. Ann. Surg..

[16] Mao T., Fang W., Gu Z., Guo X., Ji C., Chen W. (2015). Comparison of perioperative outcomes between open and minimally invasive esophagectomy for esophageal cancer. Thorac. Cancer.

[17] Mazzella A., Olland A., Falcoz P.E., Renaud S., Santelmo N., Massard G. (2016). Video-assisted thoracoscopic lobectomy: which is the learning curve of an experienced consultant?. J. Thorac. Dis..

[18] Michaelidou N., Dibb S. (2006). Using email questionnaires for research: good practice in tackling non-response. J. Target Meas. Anal. Market..

[19] Nguyen T.C., Terwelp M.D., Stephens E.H. (2015). Resident perceptions of 2-year versus 3-year cardiothoracic training programs. Ann. Thorac. Surg..

